# Apolipoprotein A1 mimetic peptide ATI-5261 reverses arterial stiffness at late pregnancy and early postpartum in a COMT^−/−^ mouse model of preeclampsia

**DOI:** 10.1186/s40885-018-0097-1

**Published:** 2018-09-15

**Authors:** Shutan Liao, Hao Wu, Ruiying Chen

**Affiliations:** 1 Rural Clinical School, University of New South Wales, Sydney, NSW Australia; 2 The First Affiliated Hospital of Sun Yat-sen University, Guangzhou, China; 3 Chashan Teaching Centre, Department of Physiology, Wenzhou Medical University, Wenzhou, 325035 Zhejiang China

**Keywords:** Apolipoprotein A1, Arterial stiffness, Mice, Preeclampsia, Cholesterol efflux, Pulse wave velocity

## Abstract

**Background:**

Preeclampsia (PE) is a serious maternal complication during pregnancy. Associated arterial stiffness in PE patients leads to increased risks of cardiovascular diseases later in life. Cholesterol efflux capacity, especially ATP binding cassette transporter A1 (ABCA1) dependent capacity, has been proposed to be a likely mediator of arterial stiffness. In the present study, we aimed to evaluate the effect of an apolipoprotein A1 mimetic peptide ATI-5261 on arterial stiffness in a mouse model of PE.

**Methods:**

Pregnant COMT^−/−^ mice were randomized to receive vehicle or ATI-5261 (30 mg/kg per day) via subcutaneous injection from gestational days (GD) 10.5 to GD 18.5 or to 10 days postpartum. Pregnant C57BL/6 J mice received vehicle during paralleled periods were served as normal controls.

**Results:**

COMT^−/−^ mice displayed maternal hypertension and proteinuria during pregnancy. Carotid–femoral pulse wave velocity (PWV) was increased at GD 18.5 and 10 days postpartum. ATI-5261 treatment in COMT^−/−^ mice significantly reduced PWV and partially normalized impaired ex vivo vascular function at late pregnancy and early postpartum. ATI-5261 treatment also increased serum ABCA1 concentrations and cholesterol efflux capacity, as well as ABCA1 expressions in the placenta. Pup weights, crown to rump lengths and abdominal circumferences were reduced in COMT^−/−^ mice. Treatment with ATI-5261 did not alter these fetal measurements but significantly reduced placental weights and increased fetal to placental ratios in COMT^−/−^ mice.

**Conclusion:**

ATI-5261 reversed arterial stiffness at late pregnancy and early postpartum in a COMT^−/−^ mouse model of PE and may be a potential therapy for arterial stiffness associated with PE.

## Background

Preeclampsia (PE), defined as the development of maternal hypertension with proteinuria after 20 weeks of pregnancy, is one of leading causes of maternal and perinatal mortality and morbidity [1, 2]. Women with PE are at increased risks of cardiovascular diseases later in life, which may be associated with persisting endothelial, cardiovascular, and metabolic dysfunction, as well as increased arterial stiffness following preeclamptic pregnancies [3–5].

Arterial stiffness, as a consequence of normal aging, endothelial dysfunction, vascular smooth muscle cell hyperplasia and inflammation, has been demonstrated to be a predictor for increased risk for cardiovascular diseases, including stroke, coronary artery disease and heart failure, independent from blood pressure [6–8]. Previous studies suggest that cholesterol efflux affects several factors that are mechanistic in arterial stiffness, such as endothelial function and inflammation [9, 10]. Therefore, cholesterol efflux has been proposed to be a likely mediator of arterial stiffness supported by increasing evidence [11]. The initial and rate-limiting step of cholesterol efflux is the transport of cellular free cholesterol to high-density lipoprotein (HDL) by cholesterol transporters, of which the best characterized are the ATP binding cassette transporter (ABC) A1, ABCG1 and scavenger receptor BI (SR-BI) [12]. As the major cell-surface transporter, ABCA1 appears to be the main contributor to arterial stiffness changes in terms of structural and cellular function. For example, ABCA1-dependent cholesterol efflux capacity is inversely associated with pulse wave velocity (PWV) in healthy subjects [13]. ABCA1 facilitates the release of prostaglandins in endothelial cells, resulting in anti-stiffening effects through vasodilation [14]. In addition, the role of ABCA1 in modulating the inflammatory response has been highlighted from animal models and humans [15].

Apolipoprotein (Apo) A1 is the major component of HDL protein that facilitates cholesterol efflux from peripheral cells through ABCA1. ATI-5261 is a 26-amino acid Apo A1 mimetic peptide designed from c-terminus of Apo E. It has been demonstrated that ATI-5261 mimics the activity of native Apo A1 and promotes cellular cholesterol efflux via the ABCA1 in vitro [16–18]. Providing the observations that decreased ABCA1-mediated cholesterol efflux capacity, as well as decreased placental expressions and serum levels of ABCA1 in PE patients [19–23], we hypothesized that ATI-5261 treatment could reverse the impaired ABCA1-dependent cholesterol efflux associated with PE and reduce arterial stiffness in this condition. Therefore, we investigated the effect of ATI-5261 treatment on arterial stiffness in a mouse model of PE.

## Methods

### Materials

ATI-5261 was synthesized (Biosynthesis Inc.) from all L-amino acids (EVRSKLEEWFAAFREFAEEFLARLKS) and capped with N-terminal acetyl and C-terminal amide groups. The lyophilized peptide (∼95% of purity) was dissolved in 10 mM phosphate buffer saline (150 mM NaCl; pH 7.4), filter sterilized (0.2 μm), and stored at 4 °C until use [24].

### Animals

This study was performed in accordance to the Guide for the Care and Use of Laboratory Animals published by the US NIH. All protocols were approved by the Animal Ethics Committee of Wenzhou Medical University (WZU38479–567). Female homozygous catechol-O-methyl transferase knockout (COMT^−/−^; Cyagen Biosciences Inc.) mice and C57BL/6 J mice (Jackson laboratories) aged 8 to 12 weeks were housed under standard conditions and maintained at 22 °C with a 12-h light/dark cycle and with ad-libitum access to food and water. Special care was taken during experimental procedures to minimize stress and discomfort to the animals. Females were mated nightly with males of a corresponding genotype and the day a vaginal plug detected was denoted as gestational day (GD) 0.5. Maternal body weight was monitored daily. Arterial blood pressure was measured in conscious mice, using the tail-cuff method before mating, at GD 10.5 and GD 18.5, and at 10 days postpartum. Mice were habituated to the restraining system for 4 consecutive days before the first measurement. ATI-5261 or vehicle was administrated via subcutaneous injection in the skinfold at the nape of neck using tuberculin syringes daily. Mice were treated with ATI-5261 at a dose of 30 mg/kg per day as one study suggests that treatment with this dose significantly reduced atherosclerosis and increased reverse cholesterol transport in Apo E^−/−^ mice [16]. Pregnant COMT^−/−^ mice (*n* = 8 per group) were randomized into 4 groups: Group A and B: mice were treated with ATI-5261 or vehicle from GD 10.5 to GD 18.5; Group C and D: mice were treated with ATI-5261 or vehicle from GD 10.5 to 10 days postpartum. Pregnant C57BL/6 J mice (n = 8 per group) also received vehicle treatment from GD 10.5 to GD 18.5 or to 10 days postpartum and were served as normal controls (designed as Group E and F). Urine samples from all groups were collected using a clear plastic wrap at GD 18.5 as previously described [25]. All mice were anaesthetized by inhalation of 5% sevoflurane and then euthanized by cervical dislocation. Mice in Group A, B and E were killed at GD 18.5 and pups and placentas dissected out. Litter sizes, pup weights and placenta weights were recorded. Fetal crown-to rump lengths and abdominal circumferences were measured. Embryonic death was determined by the presence of fetal resorption. The embryo resorption rate was calculated as the number of reabsorbed embryos/total number of embryos. Mice in Group C, D and F were culled at 10 days postpartum. Blood samples from all groups were taken by cardiac puncture and maternal livers were collected following euthanasia. Urine, serum and tissue specimens were stored at − 80 °C for further analysis.

### Pulse wave velocity assessment

Carotid–femoral pulse wave velocity (PWV) was determined noninvasively using applanation tonometry as described previously [26]. In brief, sevoflurane anaesthesia (8% and 5% in oxygen for induction and maintenance, respectively, 1.5 L/min) was used to immobilize the animals during tonometry measurements. Two pulse tonometers (SPT-301, Millar Instruments) were applanated on the skin of the right carotid and femoral arteries using a micromanipulator. The distances between the sternal notch and both measurement sites (carotid and femoral artery) were measured using a sliding caliper. Carotid–femoral transit time was determined using the time difference between the foot of carotid and femoral artery pulses. Carotid–femoral PWV was then calculated as the external carotid–femoral distance divided by transit time. PWV values of pregnant mice were recorded at GD 18.5 and 10 days postpartum.

### Wire myography

The thoracic arteries were dissected from C57BL/6 J and COMT^−/−^ mice at GD 18.5 and 10 days postpartum following euthanasia. Aortic ring segments 3–4 mm in length were prepared and wire myography was performed using an isometric wire myograph system (610 M wire myography; Danish Myo Techniques) as described previously [27]. Ex vivo vascular function was investigated by determining vasoconstrictor responses to phenylephrine (PE), endothelium-dependent relaxation, following PE pre-constriction, in response to methacholine (Mch), and endothelium-independent relaxation to the nitric oxide (NO) donor sodium nitroprusside (SNP). Finally, high potassium solution (KPSS mmol/L; 12.45 NaCl, 25 NaHCO_3_, 120 KCl, 2.4 MgSO_4_, 1.6 CaCl_2_, 1.18 KH_2_PO_4_, 5 glucose, 0.034 EDTA; pH 7.4) was added and the constriction allowed to plateau. Data were continuously collected and the vessel active wall tension (ΔT in mN/mm) was transformed to active effective pressure [ΔT/(diameter/2000)] denoted by kPa.

### Urine and blood analysis

The concentrations of urine albumin (Abcam, UK) and creatinine (CrystalChem, US), as well as serum ABCA1 and ABCG1 (LifeSpan Biosciences, US) were measured by mouse-specific enzyme-linked immunosorbent assays (ELISAs) as per the manufacturers’ instructions.

### Cholesterol efflux capacity assay

Apo B-depleted serum was prepared by addition of PEG 6000 using the method previously described [28]. Cholesterol efflux capacity assays were performed using mouse cAMP-treated J774 macrophages and several cellular models: Chinese hamster ovary (CHO)-K1, CHO-ABCG1, and CHO-ABCA1 as previously described [29, 30]. [^3^H]-cholesterol-labelled cells were incubated 4 h at 37 °C in the presence of 40-fold diluted serum samples. ABCG1-dependent efflux was calculated as the difference between efflux to CHO-ABCG1 and CHO-K1 cells. The ABCA1-dependent efflux was calculated as the difference between efflux to activated CHO-ABCA1 induced by tetracycline (1 μg/mL) and nonactivated cells. Fractional cholesterol efflux was calculated as the amount of the label recovered in the medium divided by the total label in each well. All efflux experiments were performed in triplicate for each sample.

### Western blotting

Whole-cell lysates were made from homogenized harvested maternal liver and placenta samples with radio-immunoprecipitation assay buffer (Abcam, UK) containing a protease inhibitor cocktail (Roche, UK). Protein concentrations were determined through a Direct Detect Spectrometer (Merck, Darmstadt, Germany). Western blot analysis was carried out under reducing conditions using specific antibodies (Novus Biologicals, US) to detect the expressions of ABCA1 (NB400–105), ABCG1 (NB400–132), SR-BI (NB400–104) and glyceraldehyde-3-phosphate dehydrogenase (GAPDH; NB100–56875). Immune complexes were visualized using horseradish peroxidase-conjugated secondary antibody with enhanced chemiluminescence on a BioRad Chemidoc MP system.

### Statistical analysis

All normally distributed data are expressed as means ± SEM and were compared using Student’s *t* test or one-way ANOVA with post-hoc analysis (Tukey’s procedure). Analysis was conducted using IBM SPSS Statistics 22. A *p*-value of < 0.05 was accepted as statistically significant.

## Results

### Maternal blood pressure and proteinuria

**Fig. 1:**
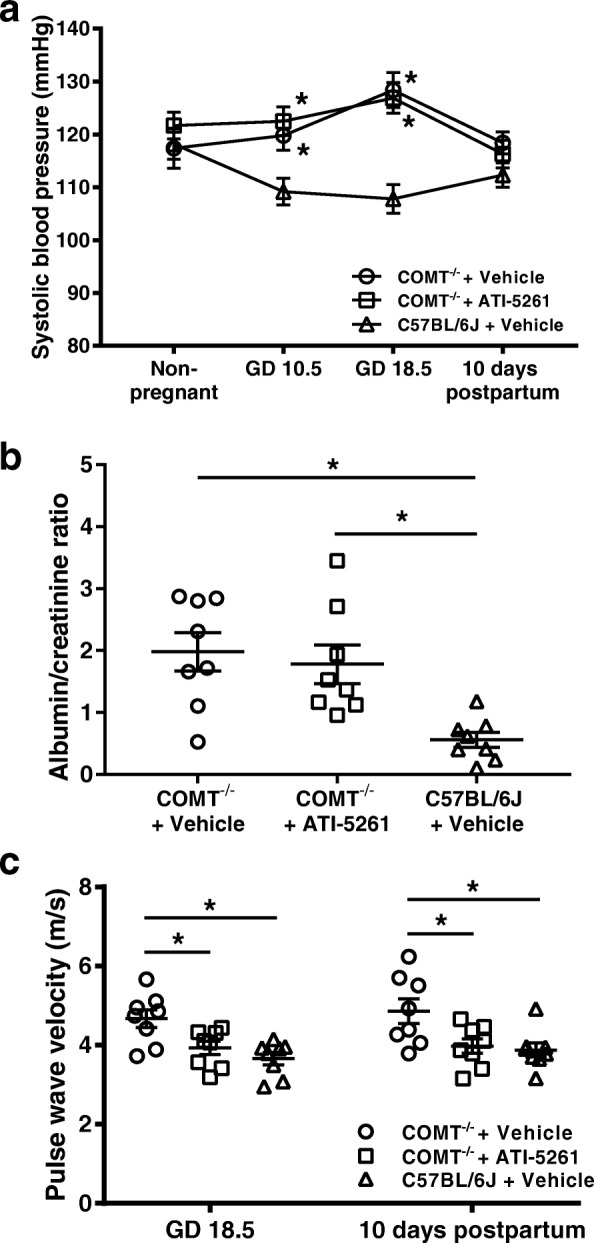
Maternal systolic blood pressure, proteinuria and pulse wave velocity. **a** Systolic blood pressure was significantly increased in vehicle treated or ATI-5261 treated COMT^−/−^ mice compared with C57BL/6 J mice at GD 10.5 and GD 18.5. ATI-5261 treatment had no effects on blood pressure at GD 18.5 or 10 days postpartum. The increased blood pressure observed in COMT^−/−^ mice returned to nonpregnant levels at 10 days after delivery. **b** Proteinuria, as assessed by albumin/creatinine ratio, was significantly increased in vehicle treated or ATI-5261 treated COMT^−/−^ mice compared with C57BL/6 J mice. Treatment with ATI-5261 had no effects on proteinuria. **c** PWV was significantly increased at GD 18.5 and 10 days postpartum in vehicle treated COMT^−/−^ mice compared with vehicle treated C57BL/6 J mice. ATI-5261 treatment significantly reduced PWV in COMT^−/−^ mice at GD 18.5 and 10 days postpartum. Data are presented as mean ± SEM. Groups (*n* = 8 in all groups) were compared using one-way ANOVA with post-hoc analysis (Tukey’s procedure). *, *p* < 0.05

Maternal blood pressure values are shown in Table 1 and Fig. 1a. Compared with nonpregnant state, systolic blood pressure in vehicle treated COMT^−/−^ mice was significantly increased at GD 18.5 and the increased blood pressure returned to nonpregnant levels at 10 days after delivery; systolic blood pressure in vehicle treated C57BL/6 J mice was significantly decreased at GD 18.5; systolic blood pressure in ATI-5261 treated COMT^−/−^ mice was unaltered during pregnancy. There were no significant differences in systolic blood pressure between nonpregnant COMT^−/−^ mice and nonpregnant C57BL/6 J mice. Systolic blood pressure was significantly increased in vehicle treated or ATI-5261 treated COMT^−/−^ mice compared with C57BL/6 J mice at GD 10.5. This increase was also observed at GD 18.5.

**Table 1:** Maternal systolic blood pressure, proteinuria and pulse wave velocity

Parameters	COMT^−/−^ mice		C57BL/6 J mice
	Vehicle (*n* = 8)	ATI-5261 (*n* = 8)	Vehicle (*n* = 8)
Systolic blood pressure (mmHg)			
Non-pregnant	117.4 ± 3.8	121.7 ± 2.5	118.2 ± 2.9
Gestational day 10.5	119.8 ± 2.8^a^	122.5 ± 2.7^a^	109.2 ± 2.5^b^
Gestational day 18.5	128.4 ± 3.3^a^	126.9 ± 2.9^a^	107.8 ± 2.7^b^
10 days postpartum	118.4 ± 2.1	116.3 ± 2.6	112.3 ± 2.3
Pulse wave velocity (m/s)			
Gestational day 18.5	4.67 ± 0.22^a^	4.02 ± 0.21^b^	3.66 ± 0.15^b^
10 days postpartum	4.86 ± 0.31^a^	3.98 ± 0.18^b^	3.88 ± 0.17^b^

Data are presented as mean ± SEM. Groups were compared using one-way ANOVA with post-hoc analysis (Tukey’s procedure). Groups which do not share the same letter are significantly different from each other

Proteinuria was assessed by calculating the albumin/creatinine ratio at GD 18.5. The ratio was significantly increased in vehicle treated or ATI-5261 treated COMT^−/−^ mice compared with vehicle treated C57BL/6 J mice (Fig. 1b). Treatment with ATI-5261 had no effects on proteinuria.

### Pulse wave velocity and arterial stiffness

Arterial stiffness was assessed by carotid–femoral PWV. Carotid–femoral PWV was significantly higher at GD 18.5 and 10 days postpartum in vehicle treated COMT^−/−^ mice compared with C57BL/6 J mice (Table 1; Fig. 1c). ATI-5261 treatment significantly reduced carotid–femoral PWV in COMT^−/−^ mice at GD 18.5 and 10 days postpartum (Table 1; Fig. 1c).

### Ex vivo vascular function

**Fig. 2:**
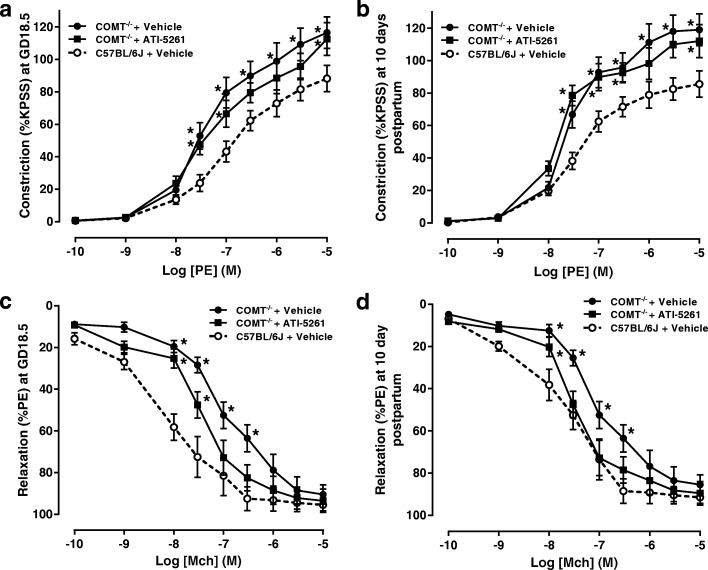
Concentration-response curves for PE or MCh in isolated aortic rings. **a** and **b** Constriction of aortic rings in response to PE was increased in vehicle treated COMT^−/−^ mice compared to C57BL/6 J mice at GD 18.5 or 10 days postpartum. **c** and **d** Relaxation of aortic rings in response to MCh was decreased in vehicle treated COMT^−/−^ mice compared to C57BL/6 J mice at GD 18.5 or at 10 days postpartum. ATI-5261 treatment partially normalized vasoconstriction and vasorelaxation in COMT^−/−^ mice to that in C57BL/6 J mice. Data are presented as mean ± SEM. Vehicle treated COMT^−/−^ mice (*n* = 10 from 5 dams), ATI-5261 treated mice (*n* = 8 from 4 dams) and C57BL/6 J mice (*n* = 10 from 5 dams) were compared using one-way ANOVA with post-hoc analysis (Tukey’s procedure). *, *p* < 0.05 (when compared with C57BL/6 J mice)

Aortic arteries from COMT^−/−^ mice exhibited increased constriction in response to PE as well as reduced relaxation in response to MCh, compared with arteries from C57BL/6 J mice (Fig. 2). After treatment with ATI-5261, artery constriction to PE in COMT^−/−^ mice at doses of 3 × 10^− 7^ M, 10^− 6^ M and 3 × 10^− 6^ M was normalized to that in C57BL/6 J mice at GD 18.5 (Fig. 2a). Similarly, ATI-5261 treatment normalized artery constriction in COMT^−/−^ mice at doses of 3 × 10^− 7^ M and 10^− 6^ M at 10 days postpartum (Fig. 2b). In terms of the endothelium-dependent relaxation response to MCh, ATI-5261 treatment normalized artery relaxation in COMT^−/−^ mice at doses of 10^− 7^ M and 3 × 10^− 7^ M at GD 18.5 and at doses of 3 × 10^− 8^ M, 10^− 7^ M and 3 × 10^− 7^ M at 10 days postpartum, respectively (Fig. 2c and d). There were no differences in relaxation in response to SNP between groups at either GD 18.5 or 10 days postpartum (data not shown).

### Fetal and placental measurements

Fetal and placental measurements are detailed in Table 2. Pup weights, crown to rump lengths and abdominal circumferences were decreased in vehicle treated COMT^−/−^ mice compared with C57BL/6 J mice. Treatment with ATI-5261 did not alter these fetal measurements in COMT^−/−^ mice. Placental weights were increased and fetal to placental ratios were decreased in vehicle treated COMT^−/−^ mice compared with C57BL/6 J mice. ATI-5261 treatment significantly reduced placental weights and increased fetal to placental ratios in COMT^−/−^ mice. There were no gross abnormalities after treatments with ATI-5261 in COMT^−/−^ mice.

**Table 2:** Fetal and placental measurements at GD 18.5

Parameters	COMT^−/−^ mice		C57BL/6 J mice
	Vehicle (*n* = 8)	ATI-5261 (*n* = 8)	Vehicle (*n* = 8)
Litter size, n	8.1 ± 0.5	8.7 ± 0.4	9.1 ± 0.6
Pup weight, mg	895.5 ± 22.8^a^	958.2 ± 19.8^a^	1225.6 ± 25.6^b^
Crown to rump length, mm	27.5 ± 0.7^a^	28.6 ± 0.5^ab^	30.4 ± 0.6^b^
Abdominal circumference, mm	26.1 ± 0.9^a^	27.7 ± 0.8^ab^	29.2 ± 0.8^b^
Placental weight, mg	98.5 ± 2.8^a^	88.9 ± 2.3^b^	85.9 ± 2.6^b^
Fetal to placental ratio	9.4 ± 0.4^a^	11.1 ± 0.4^b^	14.8 ± 0.5^c^
^#^Reabsorption rate, %	10.4	7.7	3.4

Data are presented as mean ± SEM. Groups were compared using one-way ANOVA with post-hoc analysis (Tukey’s procedure). Groups which do not share the same letter are significantly different from each other

^#^Number of reabsorbed embryos per total number of embryos in each group

### Serum ABCA1 and ABCG1

**Fig. 3:**
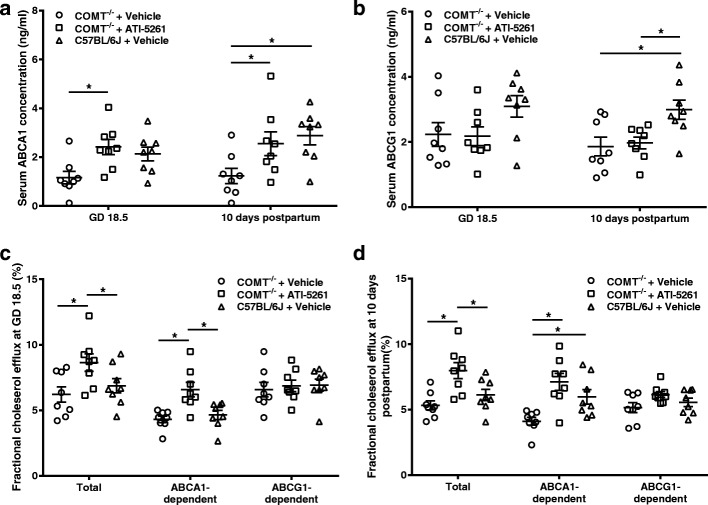
Serum ABCA1 and ABCG1 concentrations and cholesterol efflux capacity. **a** Serum ABCA1 concentrations were significantly decreased in vehicle treated COMT^−/−^ mice, compared with C57BL/6 J mice. ATI-5261 treatments increased serum ABCA1 concentrations in COMT^−/−^ mice at GD 18.5 and 10 days postpartum. **b** Serum ABCG1 concentrations were significantly decreased in vehicle treated or ATI-5261 treated COMT^−/−^ mice at 10 days postpartum. **c** and **d** Total cholesterol efflux capacity and ABCA1-dependent efflux were significantly increased in ATI-5261 treated COMT^−/−^ mice at GD 18.5 and 10 days postpartum compared to COMT^−/−^ mice. ABCA1-dependent efflux was decreased at 10 days postpartum in vehicle treated COMT^−/−^ mice compared with C57BL/6 J mice. Data are presented as mean ± SEM. Groups (n = 8 in all groups) were compared using one-way ANOVA with post-hoc analysis (Tukey’s procedure). *, *p* < 0.05

There were no significant differences in serum ABCA1 and ABCG1 concentrations at GD 18.5 between vehicle treated COMT^−/−^ mice and C57BL/6 J mice (Fig. 3a and b). However, serum ABCA1 and ABCG1 concentrations were significantly decreased in vehicle treated COMT^−/−^ mice at 10 days postpartum compared with C57BL/6 J mice (Fig. 3a and b). ATI-5261 treatment significantly increased serum ABCA1 in COMT^−/−^ mice at GD 18.5 and 10 days postpartum (Fig. 3a) but had no effects on serum ABCG1 concentrations (Fig. 3b).

### Cholesterol efflux capacity

Total cholesterol efflux capacity and ABCA1-dependent efflux were significantly increased in ATI-5261 treated COMT^−/−^ mice at GD 18.5 compared to vehicle treated COMT^−/−^ mice (Fig. 3c). The increases of total cholesterol efflux capacity and ABCA1-dependent efflux in ATI-5261 treated COMT^−/−^ mice were also observed at 10 days postpartum (Fig. 3d). Interestingly, ABCA1-dependent efflux was decreased in vehicle treated COMT^−/−^ mice at 10 days postpartum, compared to C57BL/6 J mice (Fig. 3d). ATI-5261 treatment normalized ABCA1-dependent efflux at 10 days postpartum (Fig. 3d). ABCG1-dependent efflux was not affected by mice genotypes or treatments (Fig. 3c and d).

### Cholesterol acceptor/receptor expressions

**Fig. 4:**
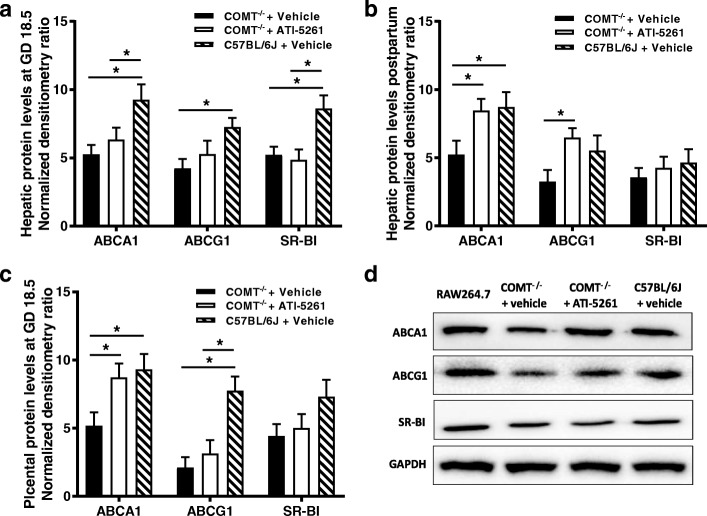
ABCA1, ABCG1 and SR-BI protein expressions. **a** and **b** Hepatic protein expressions of ABCA1, ABCG1 and SR-BI were significantly decreased in COMT^−/−^ mice at GD 18.5, compared to C57BL/6 J mice. Decreased hepatic ABCA1 expression was also observed at 10 days postpartum. ATI-5261 increased ABCA1 and ABCG1 expression in the liver at 10 days postpartum. **c** Placental protein expressions of ABCA1 and ABCG1 was reduced in COMT^−/−^ mice, compared to C57BL/6 J mice. ATI-5261 treatment significantly increased ABCA1 levels in the placenta of COMT^−/−^ mice. **d** Representative immunoblots of the corresponding proteins in the placenta with mouse RAW264.7 cell lysate included as positive control. Similar results were obtained when the experiment was repeated with lysates prepared from three batches of tissues. Data are presented as mean ± SEM. Groups (*n* = 8 in all groups) were compared using one-way ANOVA with post-hoc analysis (Tukey’s procedure). *, *p* < 0.05

Western blotting revealed that hepatic protein expressions of ABCA1, ABCG1 and SR-BI were decreased in COMT^−/−^ mice at GD 18.5, compared to C57BL/6 J mice (Fig. 4a). Decreased hepatic ABCA1 expression was also observed at 10 days postpartum (Fig. 4b). At GD 18.5, placental protein expressions of ABCA1 and ABCG1 was reduced in COMT^−/−^ mice, compared to C57BL/6 J mice (Fig. 4c and d). ATI-5261 treatment significantly increased hepatic expression of ABCA1 and ABCG1 in COMT^−/−^ mice at 10 days postpartum and increased placental expression of ABCA1 but not ABCG1 at GD 18.5 (Fig. 4).

## Discussion

In the present study, we firstly demonstrated that treatments with ATI-5261 reduced arterial stiffness at late pregnancy and early postpartum in the COMT^−/−^ mouse model of PE. ATI-5261 therapy was associated with increased serum ABCA1 concentration and cholesterol efflux capacity, as well as increased ABCA1 expression in the placenta and the liver.

The etiology of PE is currently unclear but there is a consensus that the placenta plays a key role in the pathogenesis of PE. It is believed that PE develops as consequences of impaired trophoblast invasion and inadequate remodelling of the uterine spiral arteries. Subsequent oxidative stress and inflammation in the placenta alters expressions of pro-inflammatory, anti-angiogenic and angiogenic factors, contributing to endothelial cell dysfunction and an inflammatory response [31]. The vascular dysfunction in PE patients also presents as increased arterial stiffness during pregnancy and postpartum, which is associated with inflammation in this condition [32]. However, various circulating factors that disturb the balance between vasodilatory and vasoconstrictor mechanisms are involved in the maternal vascular dysfunction of PE [33].

In the present study, we observed that systolic blood pressure was significantly decreased in C57BL/6 J mice at GD 18.5, compared with nonpregnant state. This phenomenon has also been demonstrated in previous studies [34–36]. However, the decline of maternal blood pressure was absent during COMT^−/−^ mice pregnancy. It has been postulated that altered maternal blood pressure control during pregnancy may contribute to the development of PE [37], supporting the use of pregnant COMT^−/−^ mice as an animal model of PE. Kanasaki et al. firstly demonstrated that pregnant COMT^−/−^ mice displayed a PE-like phenotype resulting from an absence of 2-methoxyoestradiol (2-ME) [38]. Following studies consistently observed maternal hypertension and increased proteinuria at late gestation, aberrant umbilical blood flow and vascular response, as wells as compromised fetal growth [39, 40]. Consistent results, particularly increased PWV and impaired vascular response, were also observed in our study. Interestingly, increased blood pressure at GD 18.5 returned to prepregnant level at 10 days after delivery. However, increased PWV persisted at 10 days postpartum. These observations suggest that persistent increased arterial stiffness may be associated with the deficiency in COMT in this mouse model of PE. 2-ME is generated by COMT in the placenta and elevated during normal human pregnancy [41, 42]. 2-ME counters angiogenesis and restores hypoxia-induced disruption by inhibiting hypoxia-inducible factor-1a [43, 44]. Emerging evidence suggests that 2-ME plays a role in cardiovascular function. It is reported that 2-ME attenuates hypertension and vascular remodelling [45], reduces atherosclerotic lesion formation [46] and inhibits neointima formation and smooth muscle cell growth [47, 48]. Moreover, 2-ME induces vasodilation by stimulating NO release in vascular endothelial cells [49], and regulates vascular tone and blood flow by inducing the synthesis of vasodilatory substances, such as NO and prostacyclin [50]. In addition, pharmacological inhibition of COMT produces arterial hypertension and endothelial dysfunction in pregnant rats which may be attributed to reduced NO bioavailability. These factors may all contribute to the increased arterial stiffness and impaired vascular function observed in the present study.

The anti-inflammatory properties of HDL and Apo A1 are well established in previous studies [51, 52]. Apo A1 mimetic peptides display anti-inflammatory and antioxidant properties and function in part by promoting cholesterol efflux and reverse cholesterol transport via ABCA1 [53, 54]. Inflammation and oxidative stress are key potential mechanisms that could induce changes in the stiffness of arteries [55, 56]. Therefore, anti-inflammatory interventions are suggested to be able to improve arterial stiffness as a potential therapy, while beneficial effects have been shown in several studies [57–59]. In the present study, ATI-5261 treatment increased serum ABCA1 concentrations and ABCA1-dependent cholesterol efflux, accompanied by reduced arterial stiffness in COMT^−/−^ mice. ABCA1 is well known for its role in regulating cholesterol transport to either cell surface-bound or internalized apolipoproteins and HDL formation [60]. Through this process ABCA1 reduces the amount of free cholesterol present in the plasma membrane and decreases membrane lipid raft content, which dampens inflammatory signalling by reducing Toll-like receptors in lipid rafts [61, 62]. The interaction of Apo A1 and ABCA1 activates Janus kinase 2/signal transducer and activator of transcription 3 (JAK2/STAT3), which suppresses pro-inflammatory genes such as tumour necrosis factor-α [63]. Macrophage-specific ABCA1 deficiency is associated with increased pro-inflammatory gene expression and cytokine release both in vivo in animal models and ex vivo [15]. The potential mechanisms are ATI-5261, as an Apo A1 mimetic peptide, functions as an anti-inflammatory agent; ABCA1 functions as an anti-inflammatory receptor to suppress the expression of inflammatory factors and to be the molecular basis for the interaction between inflammation and reserve cholesterol transport [64]. Apart from that, ABCA1 may also play an important role in regulating endothelial function [65, 66] and vascular smooth muscle cells proliferation and phenotypic changes [67], which are key factors that affect arterial stiffness.

Consistent with previous studies in humans [19, 20, 22], placental ABCA1 expressions were decreased in the COMT^−/−^ mice model of PE. The ABC transporters comprise a family of functionally diverse polytopic transmembrane proteins and are able to translocate a wide variety of substrates, including amino acids, lipids, ions, peptides and drugs, across extracellular and intracellular biological membranes [68]. In particular, ABCA1 and ABCG1 have been described to be involved in feto-maternal lipid transport and fetal lipid homeostasis [69]. Functional loss of ABCA1 in mice causes severe placental malformation and fetal growth retardation [70], also emphasizing the unique role of ABCA1 in reproduction. Therefore, the decreased fetal growth in COMT^−/−^ mice may be partially explained by the decreased levels of ABCA1 and ABCG1 in the placenta. ATI-5261 treatment significantly increased ABCA1 levels in the placenta but did not improve fetal growth in our study. The decreased placental weights and increased fetal to placental ratios by ATI-5261 treatment may indicate the improvement of placental function [71]. We speculate that the beneficial effects of ATI-5261 on placental function may not be able to encounter other deficiencies in COMT^−/−^ mice resulting in impaired fetal growth.

A limitation of this study is that only one dose of ATI-5261 had been examined. However, in vivo studies on ATI-5261 are limited. The effectiveness and safety during pregnancy still need further investigation. COMT^−/−^ mice had been used as a model of PE in the present study. Currently, a number of animal models of PE exist and have facilitated the testing of pharmacological interventions that may ameliorate or prevent PE. However, none of these animal models exactly mirror the human condition and fetal outcomes and mirror the placental pathology characterized by the impaired trophoblast invasion in pregnancies complicated with PE [72].

## Conclusions

This is the first study, to our knowledge, to investigate the effect of ATI-5261 treatment on arterial stiffness in a mouse model of PE. In conclusion, ATI-5261 reversed arterial stiffness at late pregnancy and early postpartum in COMT^−/−^ mice. This important study provides crucial evidence to support the further research and development of a potential new therapy for arterial stiffness associated with PE.

## Abbreviations

2-ME: 2-methoxyoestradiol
ABCA1: ATP binding cassette transporter A1
ABCG1: ATP binding cassette transporter G1
Apo: Apolipoprotein
COMT: Catechol-O-methyl transferase
GAPDH: Glyceraldehyde-3-phosphate dehydrogenase
GD: Gestational day
HDL: High-density lipoprotein
PE: Preeclampsia
PWV: Pulse wave velocity
SR-BI: Scavenger receptor BI
